# Neuroimaging Epicenters as Vulnerable Nodes in Plasma p-tau217/Aβ42-Positive Alzheimer’s Disease

**DOI:** 10.34133/research.1392

**Published:** 2026-08-24

**Authors:** Qian Li, Yiyang Liu, Qian Chen, Xin Li, Yajing Zhu, Qiming Deng, Xin Zhang, Futao Chen, Yi Zhang, Danni Ge, Zhuoru Jiang, Xi Wu, Wen Zhang, Jiaming Lu, Bing Zhang

**Affiliations:** ^1^ Department of Radiology, Nanjing Drum Tower Hospital, Affiliated Hospital of Medical School, Nanjing University, Nanjing, China.; ^2^ Department of Psychology, University of Illinois Urbana-Champaign, Champaign, IL, USA.; ^3^ Medical Imaging Center, Nanjing Drum Tower Hospital, Affiliated Hospital of Medical School, Nanjing University, Nanjing, China.; ^4^ Institute of Medical Imaging and Artificial Intelligence, Nanjing University, Nanjing, China.; ^5^ Institute of Brain Science, Nanjing University, Nanjing, China.

## Abstract

Plasma p-tau217/Aβ42 accurately captures the systemic molecular risk of Alzheimer’s disease (AD) but lacks the spatial resolution necessary to predict individualized clinical trajectories. Here, we combined plasma biomarker stratification with normative connectome mapping to identify macroscale neurodegenerative epicenters and developed a personalized prognostic tool, the Network Vulnerability Index (NVI). Across the Alzheimer’s Disease Neuroimaging Initiative and independent China ADNI cohorts, plasma p-tau217/Aβ42-positive individuals exhibited highly reproducible epicenters tightly anchored to the default mode network and limbic axis. Multiscale analyses revealed that this spatial vulnerability aligned with transcriptomic signatures of synaptic and mitochondrial dysfunction, monoaminergic receptor density gradients, and memory-related cognitive domains. Longitudinally, baseline epicenter centrality strictly dictated future localized atrophy rates. To translate these group-level topological constraints into a personalized prognostic metric, we utilized least absolute shrinkage and selection operator regression to formulate the NVI. Cross-sectionally, the NVI robustly tracked progressive tau-positron emission tomography accumulation (meta-temporal *r* = 0.547) and hippocampal atrophy (*r* = −0.455). Crucially, the NVI demonstrated robust, stage-dependent prognostic utility. When evaluated across the continuous disease spectrum, incorporating the NVI into a fully adjusted baseline model comprising plasma p-tau217/Aβ42 and *APOE*-ε4, and clinical scores significantly improved the prediction of conversion from mild cognitive impairment to dementia (hazard ratio = 1.47, *P* = 0.004), providing essential incremental prognostic value. Collectively, our spatially contextualized framework demonstrates that mapping systemic molecular risk onto structural network vulnerability supports a highly scalable “plasma pre-screen plus standard MRI” triage pathway for precision staging in Alzheimer’s disease.

## Introduction

Accurate early detection and precise prognostic staging in Alzheimer’s disease (AD) have become a central focus of current research, as emerging disease-modifying therapies require reliable stratification before irreversible neurodegeneration occurs [1–3].

Blood-based biomarkers have recently transformed the diagnostic landscape by providing cost-efficient and minimally invasive indices of core AD pathology [4–6]. Among these, the plasma phosphorylated tau (p-tau) 217 to amyloid-β (Aβ) 42 ratio has emerged as a robust and scalable biomarker that improves diagnostic accuracy, disease staging, and prediction of clinical progression across the AD continuum [7–11], aligning with the recent shift toward biologically defined diagnostic criteria [6]. Recent studies indicate that plasma p-tau217/Aβ42 ratio is particularly advantageous for both early detection and continuous disease staging. While p-tau217 detects initial pathology even before amyloid-positron emission tomography (PET) positivity and increases dynamically, Aβ markers often plateau early [12,13]. Combining them therefore captures a broader pathological window. Crucially, the p-tau217/Aβ42 ratio effectively mitigates peripheral confounders (such as renal or metabolic dysfunction) that frequently bias absolute p-tau217 levels [7,9,14–16], yielding a highly stable and specific measure of AD-related neuropathology. However, while the p-tau217/Aβ42 ratio reliably indicates the presence and severity of AD pathology, it provides limited information regarding where neurodegeneration is most likely to initiate or how vulnerability propagates across large-scale networks.

Converging evidence from neuroimaging and postmortem studies suggests that AD progression is fundamentally constrained by the intrinsic architecture of the human connectome. Foundational studies have demonstrated that neurodegenerative syndromes predictably target specific, large-scale intrinsic networks [17], with neuropathology (particularly tau tangles) emerging in selective regional “epicenters” and propagating transneuronally along highly connected functional and structural pathways [18]. Consequently, mapping the network-based epicenters offers a principled approach to decode the spatial temporal dynamics of the disease [19]. By aligning individual patterns of gray matter variation with normative connectivity, epicenter mapping identifies critical regions whose intrinsic network topology best explains system-level neurodegeneration.

However, despite the explanatory power of connectome-based disease modeling, substantial gaps remain. First, previous network propagation models have predominantly relied on costly or invasive PET and cerebrospinal fluid (CSF) markers to stage the disease, leaving it unclear how the highly accessible, ultra-early molecular risk captured by blood biomarkers translates into macroscopic structural breakdown. Second, while existing studies have successfully mapped epicenters at the group level [19,20], there is a pressing need to translate these topological patterns into individualized prognostic indices. Bridging this gap is crucial, as identifying these structural “hotspots” within the connectome would not only provide a spatial anchor for molecular risk detected by blood assays but also forecast the specific trajectory of subsequent atrophy and clinical decline [17,18,21].

Building on this foundation, we aimed to determine whether individuals positive for the plasma p-tau217/Aβ42 ratio exhibit distinct epicenters of neuroanatomical vulnerability. Specifically, we (a) identified a convergent, group-level epicenter map in the Alzheimer’s Disease Neuroimaging Initiative (ADNI) cohort and validated its reproducibility in an independent sample; (b) examined whether this epicenter map converged with independent molecular, neurotransmitter, and cognitive architectures; (c) tested whether the baseline epicenter map predicted the spatial pattern of future longitudinal atrophy; (d) derived an individualized Network Vulnerability Index (NVI) and biologically validated it against core Alzheimer’s disease markers (Tau-PET, Aβ-PET, and hippocampal volume); and (e) evaluated the prognostic value of the NVI for predicting longitudinal clinical conversion. Through this integrative approach, we position epicenter mapping within the AD biomarker continuum, providing a spatially resolved, biologically validated, and clinically actionable framework for understanding network-constrained disease progression.

## Results

### Sample characteristics

As shown in Table 1, the ADNI discovery cohort included 573 individuals (312 negative and 261 positive) after excluding participants with intermediate p-tau217/Aβ42 ratio values. Prior to demographic comparisons, a cross-sectional validation against baseline Aβ-PET imaging (*n* = 582, encompassing the positive, intermediate, and negative biomarker groups) confirmed that the p-tau217/Aβ42 ratio (area under the curve [AUC] = 0.910) significantly outperformed p-tau217 alone (AUC = 0.890; DeLong’s test, *P* = 0.0003; Fig. S1) in dichotomizing participants by AD pathology, thereby reinforcing the validity of the applied stratification.

**Table 1.** Demographic and clinical characteristics. Data obtained from ADNI are presented in italics. Continuous variables are reported as median (interquartile range *[Q1, Q3]*); categorical variables as *n* (%). Two-sided tests: Mann–Whitney U for continuous variables; χ² (or Fisher’s exact when expected counts <5) for categorical variables.

	ADNI			C-ADNI		
Characteristics	Ratio-negative ^a^, *N* = 312	Ratio-positive ^a^, *N* = 261	*P*	Ratio-negative ^a^, *N* = 184	Ratio-positive ^a^, *N* = 58	*P*
Age, year	*69.7 (65.9, 74.5)*	*74.6 (70.3, 79.5)*	*<0.001* ^*b*^	67 (60, 70.25)	67 (63.3, 74.8)	0.053
Female sex, *n* (%)	*180 (57.69%)*	*118(45.21%)*	*0.003* ^*b*^	118 (64.13%)	40 (68.97%)	0.606
Education, year	*16.00 (14.25, 18.00)*	*16.00 (14.00, 18.00)*	*0.091*	12 (10.75, 15)	12 (10, 15)	0.827
*APOE-ε4* carriers^c^, *n* (%)	*71 (22.83%)*	*176 (67.69%)*	*<0.001* ^*b*^	15 (8.2%)	19 (32.8%)	<0.001 ^*b*^
Cognitive states, *n* (%)	*<0.001* ^*b*^			0.366
CU	*226 (72.44%)*	*74 (28.35%)*		48 (26.1%)	15 (25.9%)	
SCD	*-*	*-*		80 (43.5%)	23 (39.7%)	
MCI	*81 (25.96%)*	*117 (44.82%)*		55 (29.9%)	18 (31%)	
Dementia	*5 (1.60%)*	*70 (26.82%)*		1 (0.5%)	2 (3.4%)	
Cognitive scales						
CDR-SB	*0.00 (0.00, 5.00)*	*1.50 (0.00, 3.00)*	*<0.001* ^*b*^	-	-	-
MMSE	*29.00 (28.00, 30.00)*	*27.00 (24.50, 29.00)*	*<0.001* ^*b*^	29 (28, 30)	28 (27, 29)	0.114
MoCA	*26.00 (23.00, 28.00)*	*22.00 (18.00, 25.00)*	*<0.001* ^*b*^	25 (23, 27)	24 (22, 27)	0.23

ADNI, Alzheimer’s Disease Neuroimaging Initiative; C-ADNI, China Alzheimer’s Disease Neuroimaging Initiative. Cognitive states: CU, cognitively unimpaired; SCD, subjective cognitive decline; MCI, mild cognitive impairment. Cognitive scales: CDR-SB, Clinical Dementia Rating–Sum of Boxes (higher scores indicate worse function); MMSE, Mini-Mental State Examination (0 to 30); MoCA, Montreal Cognitive Assessment (0 to 30)

^a^ “Ratio-positive/negative” denotes classification by plasma p-tau217/Aβ42 (cutoffs defined in Materials and Methods; participants with intermediate values were excluded from the main analyses).

^b^ *P* ≤ 0.05.

^c^ *APOE-ε4* carriers: individuals with ≥1 ε4 allele. *APOE* genotype data were missing for 2 individuals in the ADNI discovery cohort (1 positive, 1 negative) and 51 individuals in the C-ADNI validation cohort (7 positive, 44 negative).

Compared with the negative group, p-tau217/Aβ42 ratio-positive participants were significantly older (median 74.6 vs. 69.7 years, *P* < 0.001), less frequently female (45.2% vs. 57.7%, *P* = 0.003), and had a higher frequency of *APOE-ε4* carriers (67.7% vs. 22.8%, *P* < 0.001), with no group difference in years of education. Cognitive status distribution differed markedly between groups (*P* < 0.001); the negative group consisted predominantly of cognitively unimpaired (CU) individuals (226, 72.44%), with smaller proportions of non-AD mild cognitive impairment (MCI, 81, 25.96%) and non-AD dementia (5, 1.60%), whereas the positive group was composed of a more distributed mix of CU (74, 28.35%), MCI due to AD (117, 44.82%), and AD dementia (70, 26.82%) participants. Consistent with this, cognitive scores indicated greater impairment in the positive group across all scales, including higher Clinical Dementia Rating–Sum of Boxes (CDR-SB) and lower Mini-Mental State Examination (MMSE) and Montreal Cognitive Assessment (MoCA) scores (all *P* < 0.001). The independent validation cohort (China ADNI [C-ADNI]; *n* = 242; 184 negative, 58 positive) exhibited broadly comparable demographic and clinical patterns, similarly showing a significantly higher proportion of *APOE*-ε4 carriers in the positive group (8.2% vs. 32.8%, *P* < 0.001).

### Epicenter identification and validation

**Fig. 1.**
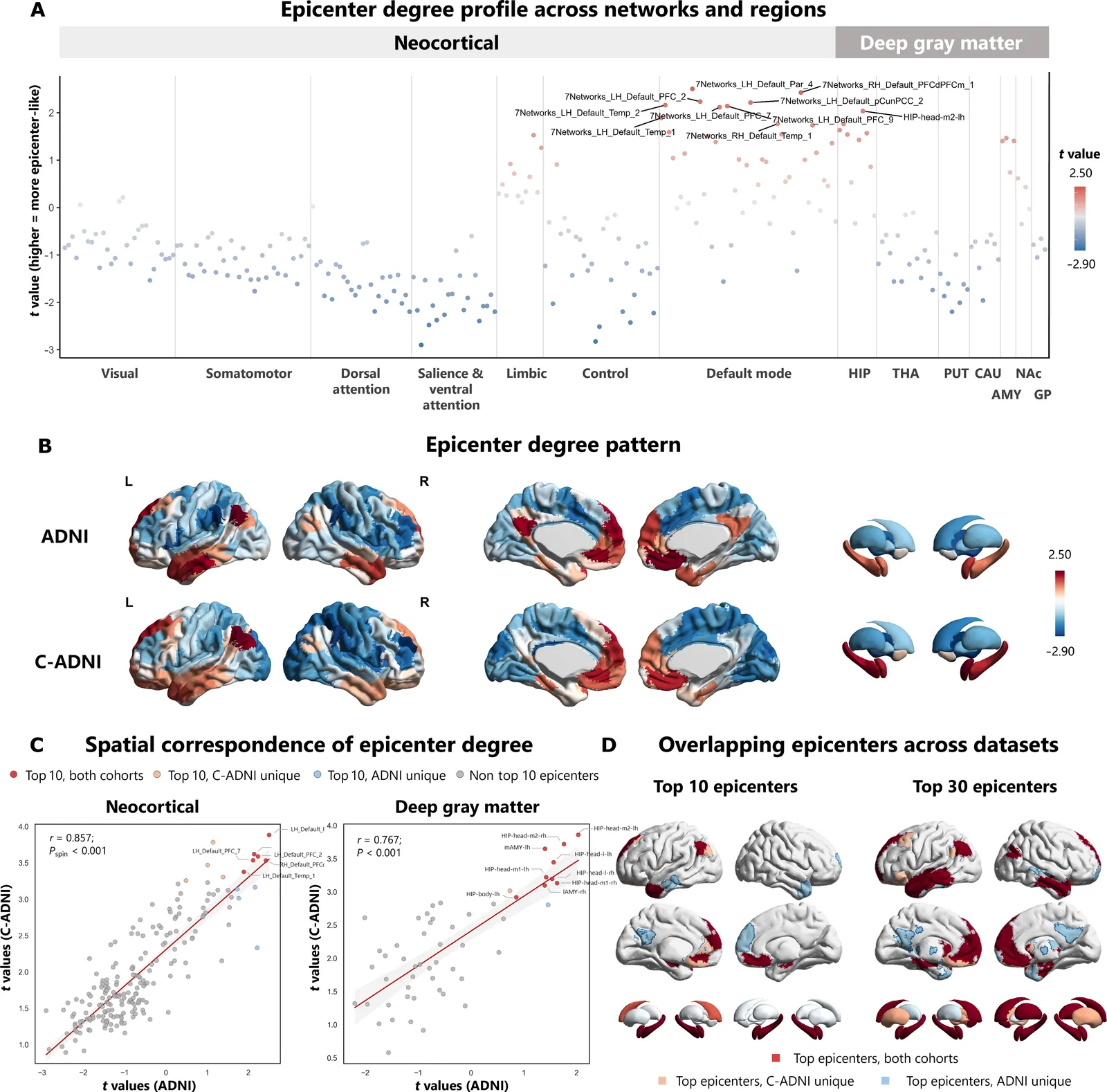
Identification and validation of top epicenter regions. (A) Manhattan plot of epicenter *t* values across 254 brain regions derived from ADNI cohort. The plot displays the *t* values of each brain region, which were derived from the network-level “goodness-of-fit” (GOF) calculations. Each region is colored according to its associated network, with cortical regions categorized into the Visual, Somatomotor, Dorsal Attention, Salience and Ventral Attention, Limbic, Control, Default Mode, and Deep Gray Matter networks. The deep gray matter structures are further divided into the following substructures: HIP (hippocampus), THA (thalamus), PUT (putamen), CAU (caudate), AMY (amygdala), NAc (nucleus accumbens), and GP (globus pallidus). The *t* values represent the strength of the epicenter association, with higher *t* values indicating stronger association with the epicenter in the network. Points labeled represent the top 10 regions across the entire brain. (B) Epicenter *t*-maps derived from 2 cohorts: ADNI discovery sample (left) and the China ADNI (C-ADNI) cohort (right), shown on the same color scale. (C) Spatial correspondence of epicenter degree across discovery and validation cohorts. Scatter plots depict the relationship between regional epicenter degree values derived from the discovery cohort (ADNI; x-axis) and the C-ADNI validation cohort (y-axis), separately for cortical (*n* = 200) and deep gray matter structures (*n* = 54). Red points indicate overlapping top 10 epicenter regions in both cohorts, blue points denote regions ranked in the top 10 only in the ADNI cohort, and salmon pink points denote regions ranked in the top 10 only in the validation cohort. (D) Spatial overlap of top epicenter regions between the discovery and validation cohorts. Overlapping epicenter regions were identified by ranking regional epicenter degree within each cohort and comparing the top regions. Panels display overlap maps for the top 10 and top 30 regions, separately for cortical and deep gray matter structures. Regions unique to the ADNI epicenter map are shown in blue, regions unique to the validation epicenter map in salmon pink, and overlapping regions in red. Notably, the highest-overlap regions were consistently localized to the default-mode network (medial prefrontal, posterior cingulate, and temporal cortices) and hippocampal structures, indicating robust spatial reproducibility of epicenter localization across independent datasets.

Within the p-tau217/Aβ42 ratio-positive group, regions exhibiting the highest epicenter degree were predominantly located within the default mode network (DMN) and limbic structures (Fig. 1A). The top neocortical epicenters included the medial prefrontal cortex, posterior cingulate/precuneus, and lateral temporal regions, whereas the leading deep gray matter epicenters were observed in the hippocampal head and body as well as the amygdala bilaterally. Together, these regions form a core vulnerability pattern along the DMN–limbic network axis, a network consistently implicated in early AD pathology. The topographical distribution of these core epicenters was exceptionally robust across a wide spectrum of alternative voxel-selection thresholds (z > 0.3, 0.4, 0.6, and 0.7; all Spearman *ρ* > 0.99, *P* < 0.001; Fig. S2).

Validation in the C-ADNI cohort demonstrated high spatial correspondence with the ADNI-derived epicenter degree map (Fig. 1B). Regional *t* values showed strong neocortical concordance (Pearson *r* = 0.857, *P* = 5.96 × 10^−59^; *P*_spin_ < 0.001) and robust deep gray matter agreement (Pearson *r* = 0.767, *P* = 1.38 × 10^−11^; Fig. 1C). Overlap of top-ranked regions was substantial: at the top 5 threshold, 2/5 neocortical and 3/5 deep gray matter epicenters overlapped; at the top 10 threshold, 6/10 neocortical and 9/10 deep gray matter epicenters overlapped; at the top 30 threshold, 22/30 neocortical and 22/30 deep gray matter epicenters overlapped (Fig. 1D).

Additionally, replicating the analysis with the Brainnetome Atlas yielded consistently anchored epicenters within the DMN and limbic network (Fig. S3). Furthermore, to account for aging-related network dedifferentiation, a sensitivity analysis using a cohort-specific normative connectome confirmed the robust preservation of the epicenter spatial topography across the whole brain (Spearman *ρ* = 0.410, *P* < 0.001; Fig. S4). Collectively, these results confirm that the network-based epicenter pattern is highly reproducible across independent samples, platforms, parcellation resolutions, analytical thresholds, and reference normative connectomes, with the most vulnerable nodes consistently aligning along the DMN–limbic network axis.

### Multiscale associations of neuroimaging epicenters

#### Transcriptomic associations

Across 254 regions and 15,633 genes, 2 partial least squares (PLS) components were estimated. PLS1 explained 1.83% of imaging variance and 0.97% of transcriptomic variance, with a cross-block correlation of *r* = 0.71. PLS2 accounted for 0.76% of imaging variance and 1.77% of transcriptomic variance. Spin permutation tests confirmed the spatial significance of PLS1 (*P*_spin_ < 0.001), and subsequent analyses therefore focused on this component. Bootstrap resampling yielded stable gene weights, which were ranked by bootstrap-derived *z*-scores and subjected to preranked gene set enrichment analysis (Fig. S5A). Among Gene Ontology (GO) terms, 62 survived false discovery rate (FDR) correction (*q* < 0.05). Notably, cellular component terms were heavily centered on synaptic elements, with the postsynaptic density membrane term exhibiting prominent enrichment (|NES| > 2.50), driven by leading-edge genes such as *DLG2*, *PRR7*, *NETO1*, and *GRIA1*. Additionally, 5 Kyoto Encyclopedia of Genes and Genomes pathways passed FDR correction (*q* < 0.05), highlighting mitochondrial electron transport and thermogenesis, with key leading-edge drivers including *NDUFB9*, *COX14*, *NDUFA12*, and *NDUFA6*. Collectively, these cross-modal analyses identify a spatial alignment between epicenter vulnerability and normative molecular gradients involved in synaptic organization and metabolic pathways.

#### Neurotransmitter receptor associations

Spatial correspondence between the epicenter *t*-map and in vivo neurotransmitter receptor density maps revealed significant associations across 3 systems (Fig. S5B). After spin permutation correction (*P*_spin_ < 0.05), epicenter *t* values were negatively correlated with dopamine D_2_ receptor availability (*r* = −0.47, *P*_spin_ < 0.002), noradrenaline transporter (*r* = −0.41, *P*_spin_ < 0.003) and 5-HT_1B_ receptor maps (*r* = −0.28, *P*_spin_ < 0.05). These findings suggest that regions most vulnerable to atrophy are preferentially aligned with spatial gradients of dopaminergic, noradrenergic, and serotonergic signaling.

#### Cognitive process associations

To situate epicenter vulnerability within large-scale functional architectures, we examined its spatial correspondence with 115 meta-analytic cognitive association maps from Neurosynth. PLS analysis identified the PLS1, which explained a substantial proportion of variance in the cognitive matrix and correlated strongly with the epicenter distribution (*r* = 0.73, *P*_spin_ = 0.002). Bootstrap analysis revealed 70 cognitive terms surviving FDR correction (*q* < 0.05). The strongest positive loadings involved declarative and autobiographical memory (e.g, semantic and episodic memory) and socio-affective processes (e.g., social cognition, valence, and familiarity), whereas negative loadings were linked to executive control, action, and sensorimotor functions (e.g., working memory, planning, and movement; Fig. S5C). Collectively, these multiscale analyses demonstrate that the identified epicenter network aligns with molecular signatures of synaptic and metabolic activity, neurotransmitter gradients involving dopamine and serotonin systems, and functional domains supporting memory and socio-affective cognition.

### Atrophy dynamics across p-tau217/Aβ42-defined groups

**Fig. 2.**
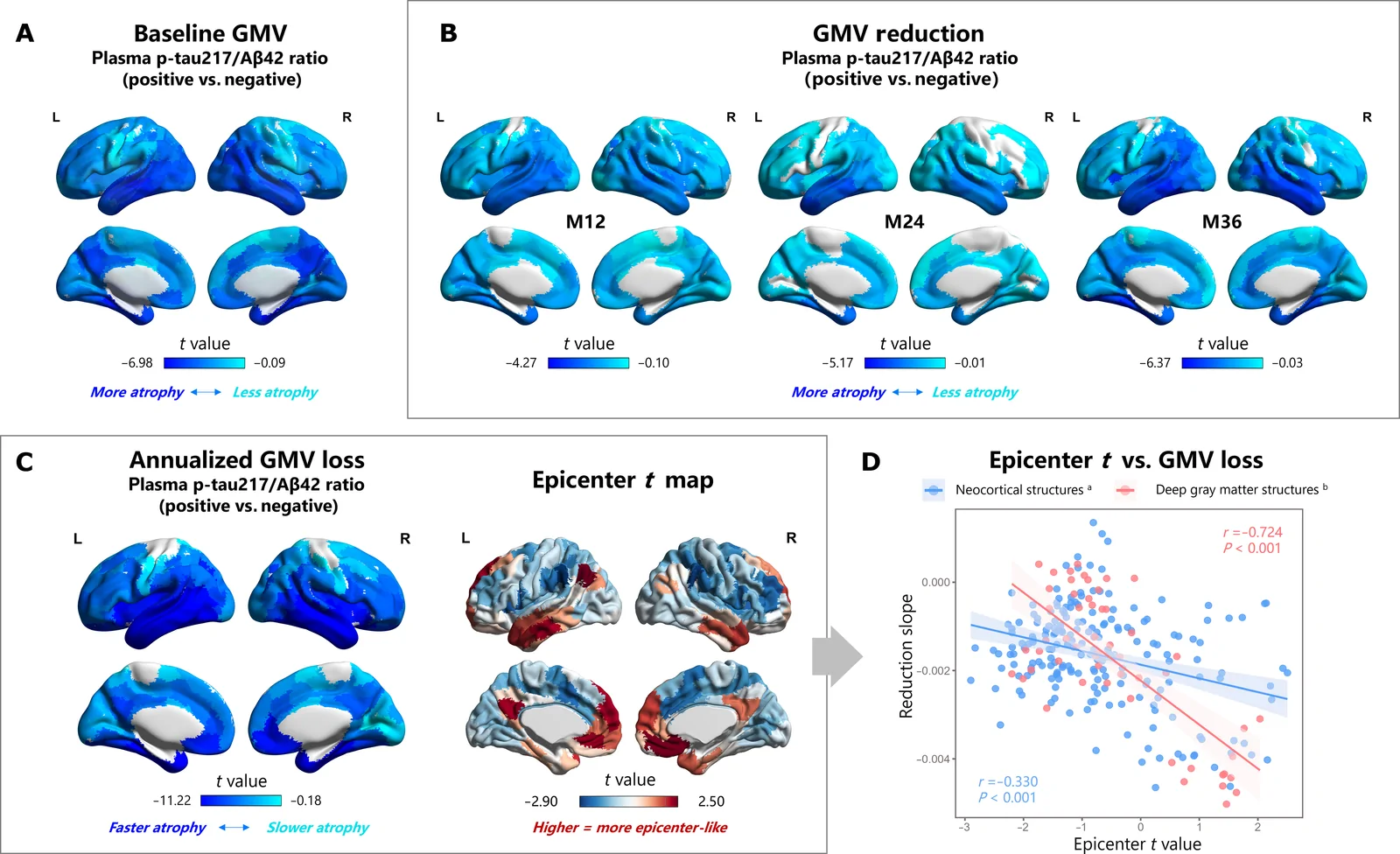
Longitudinal gray matter atrophy patterns and their spatial association with the epicenter map. (A) Cortical surface projection of baseline gray matter volume (GMV) differences between plasma phosphorylated tau (p-tau) 217/Aβ42-positive and p-tau217/Aβ42-negative groups. (B) Cortical regional GMV change at fixed follow-up intervals (12, 24, and 36 months), illustrating consistently greater cumulative atrophy in the plasma p-tau217/Aβ42-positive group. (C) Cortical group-difference map of annualized GMV loss (positive minus negative). The baseline cortical epicenter *t*-map is displayed alongside for visual comparison. (D) Scatterplot linking regional epicenter *t* values (epicenter centrality) to annualized GMV loss across neocortical (sky-blue fit) and deep gray matter (pink fit) structures; higher epicenter centrality tracks faster longitudinal GMV decline. ^a^ Neocortical and ^b^ deep gray matter structures were defined using the Schaefer 200-parcel and Tian 54-parcel atlases, respectively. Corresponding deep gray matter atrophy maps are detailed in Fig. S6.

Baseline comparisons revealed that individuals in the p-tau217/Aβ42 ratio-positive group exhibited significantly lower gray matter volume (GMV) compared with the negative group (Fig. 2A). Extending this to longitudinal intervals, the neurodegeneration observed in the positive group was consistent across fixed follow-up time points (12, 24, and 36 months), indicating a sustained pathological trajectory (Fig. 2B).

Quantifying the rate of decline via annualized atrophy slopes confirmed significantly accelerated neurodegeneration in the positive group, manifesting as a distributed yet convergent vulnerability pattern (Fig. 2C). The most severely affected regions were predominantly located in the temporal and limbic cortices (e.g., temporal pole, parahippocampal cortex, prefrontal cortex, and default-mode temporal subregions), as well as bilateral hippocampal subfields and amygdalar nuclei. Additional atrophy was observed in posterior default-mode, orbitofrontal and salience/ventral attention regions, reflecting a network-specific progression.

Region-level analyses confirmed a strong spatial relationship between baseline epicenter centrality and subsequent atrophy rates. Across cortical regions, atrophy slope correlated negatively with epicenter *t* values (*r* = −0.330, *P* < 0.001), with an even stronger association in subcortical structures (*r* = −0.724, *P* < 0.001). These findings indicate that regions identified as epicentral at baseline undergo the most rapid structural decline, underscoring the specific vulnerability of DMN–limbic circuits to longitudinal degeneration (Fig. 2D).

### Data-driven formulation and external validation of the NVI

**Fig. 3.**
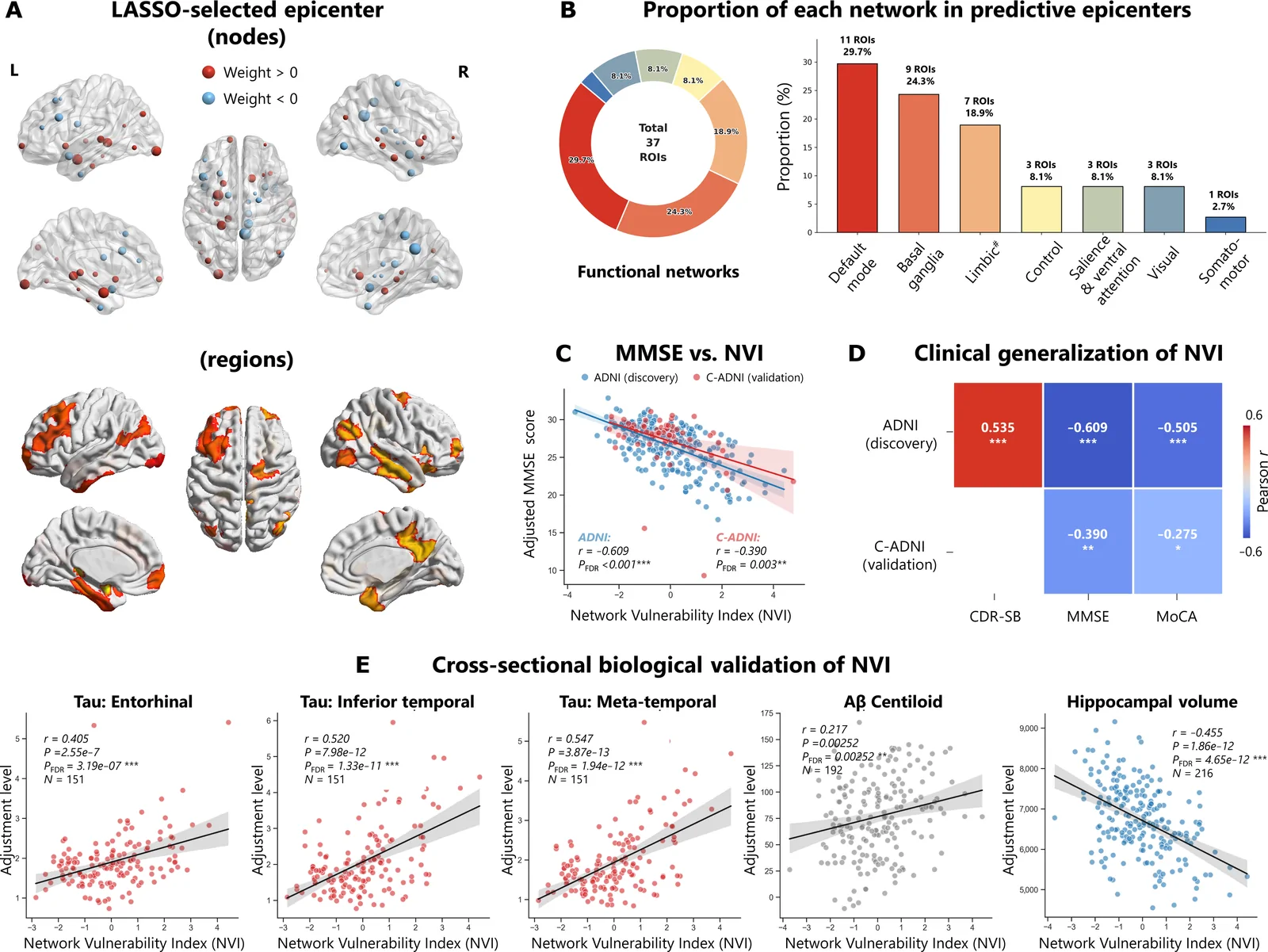
Data-driven identification and clinical validation of the predictive epicenter network. (A) Spatial distribution of the 37 predictive epicenters identified by the least absolute shrinkage and selection operator (LASSO) model. The top brain maps display the nodal representation, where node size reflects the absolute weight of the LASSO coefficient, and color indicates the directionality (red, positive weights; blue, negative weights). The bottom brain maps illustrate the corresponding anatomical regions of these epicenters. (B) Donut and bar charts detailing the proportional distribution of the 37 predictive regions across functional networks. The default mode network, basal ganglia, and limbic network emerged as the most predominant systems within this vulnerable configuration. # indicates that the medial temporal lobe structures (hippocampus and amygdala) were integrated into the limbic network for this analysis. (C) Scatter plots demonstrating the association between the Network Vulnerability Index (NVI) and age-, sex-, and education-adjusted Mini-Mental State Examination (MMSE) residuals within the plasma-positive subcohorts in both the ADNI discovery cohort and the independent China ADNI (C-ADNI) validation cohort. (D) Heatmap displaying the Pearson correlation coefficients (*r*) between the NVI and multidimensional cognitive assessments across the plasma-positive individuals in both cohorts. (E) Cross-sectional biological validation of the NVI against core A/T/N biomarkers within the plasma phosphorylated tau (p-tau) 217/Aβ42 ratio-positive subcohort. For panels (D) and (E), all *P* values were adjusted for multiple comparisons using the false discovery rate procedure.

We employed a least absolute shrinkage and selection operator (LASSO) regression model within the plasma p-tau217/Aβ42 ratio-positive ADNI discovery cohort. Using 10-fold cross-validation, the model penalized collinearity and selected features to predict baseline cognitive performance (age-, sex-, and education-adjusted MMSE residuals) based on whole-brain regional “goodness-of-fit” (GOF) scores. The LASSO algorithm shrank uninformative regional coefficients to zero, yielding a predictive network comprising 37 distinct brain regions (Fig. 3A and Table S1). Anatomical profiling of this multivariate signature revealed that the selected nodes were predominantly anchored within the DMN, basal ganglia, and the limbic system, which collectively accounted for the majority of the predictive features (Fig. 3B).

Based on the nonzero coefficients derived from this model, we formulated a composite metric termed the NVI. For each individual, the NVI was calculated as the linear combination of their regional GOF scores weighted by the LASSO-derived *β* coefficients. To align the interpretability of this index with clinical intuition, the final scores were multiplied by −1, such that a higher NVI value reflects greater spatial vulnerability and more severe network impairment.

To assess the external generalizability of the index, we performed an out-of-sample validation. The computational pipeline and model parameters derived from the ADNI cohort, including the standard scalers, confounder regression weights, and the 37 spatial LASSO coefficients, were applied directly to the independent C-ADNI validation cohort. The ADNI-trained NVI significantly predicted the adjusted MMSE residuals in the unseen C-ADNI cohort (ADNI: *r* = −0.609, *P*_FDR_ = <0.001; C-ADNI: *r* = −0.390, *P*_FDR_ = 0.003; Fig. 3C). Furthermore, the NVI demonstrated significant correlations with multiple other cognitive assessments across both cohorts (*|r|* = 0.275 to 0.609, *P*_FDR_ < 0.05; Fig. 3D). This external validation indicates that the NVI captures a generalizable spatial vulnerability pattern, functioning as a quantitative macroscale proxy for individual disease severity.

### Cross-sectional biological validation of NVI

To elucidate the biological underpinnings of the network vulnerability pattern, we evaluated the cross-sectional associations between the NVI and established multidimensional biomarkers within the A/T/N (Amyloid/Tau/Neurodegeneration) framework. Given that the NVI signature was originally derived from the plasma p-tau217/Aβ42 ratio-positive individuals, we directly evaluated its cross-sectional associations within this specific subcohort.

Consistent with the spatial distribution of the epicenter network, the NVI exhibited robust positive correlations with regional tau-PET tracer uptake (Fig. 3E). The associations were highly significant across progressive topographic stages of tau pathology, including the entorhinal cortex (*r* = 0.405, *P*_FDR_ < 0.001), inferior temporal cortex (*r* = 0.520, *P*_FDR_ < 0.001), and the broader meta-temporal region (*r* = 0.547, *P*_FDR_ < 0.001). Conversely, the association with global amyloid burden (Aβ Centiloid) was significant but modest in effect size (*r* = 0.217, *P*_FDR_ = 0.0025). This modest correlation aligns with the biological trajectory of AD, as amyloid accumulation typically plateaus in plasma p-tau217/Aβ42-positive individuals, whereas tau pathology and network structural alterations continue to progress synergistically.

Furthermore, serving as a macroscopic index of structural impairment, the NVI demonstrated a strong negative correlation with hippocampal volume (*r* = −0.455, *P*_FDR_ < 0.001). Collectively, these findings (Fig. 3E) confirm that the mathematical NVI formulation is not merely a topological abstraction but rather a faithful macroscopic proxy for the underlying cascade of molecular tau spreading and subsequent localized neurodegeneration.

### Stage-dependent prognostic utility and temporal predictive accuracy of the NVI

We next evaluated the clinical utility of the NVI for predicting longitudinal disease progression. To rigorously isolate the prognostic value of neuroimaging-derived network vulnerability from the confounding effects of baseline amyloidosis, these survival analyses were conducted strictly within the plasma p-tau217/Aβ42 ratio-positive subcohort. For this longitudinal evaluation, we tracked individuals at risk of clinical progression, encompassing 66 CU participants (42 incident conversions) and 95 individuals with MCI (53 incident conversions). We employed multivariate Cox proportional hazards models, adjusting for age, sex, education, and *APOE*-ε4 status, alongside time-dependent receiver operating characteristic (ROC) analyses to assess temporal predictive accuracy.

**Fig. 4.**
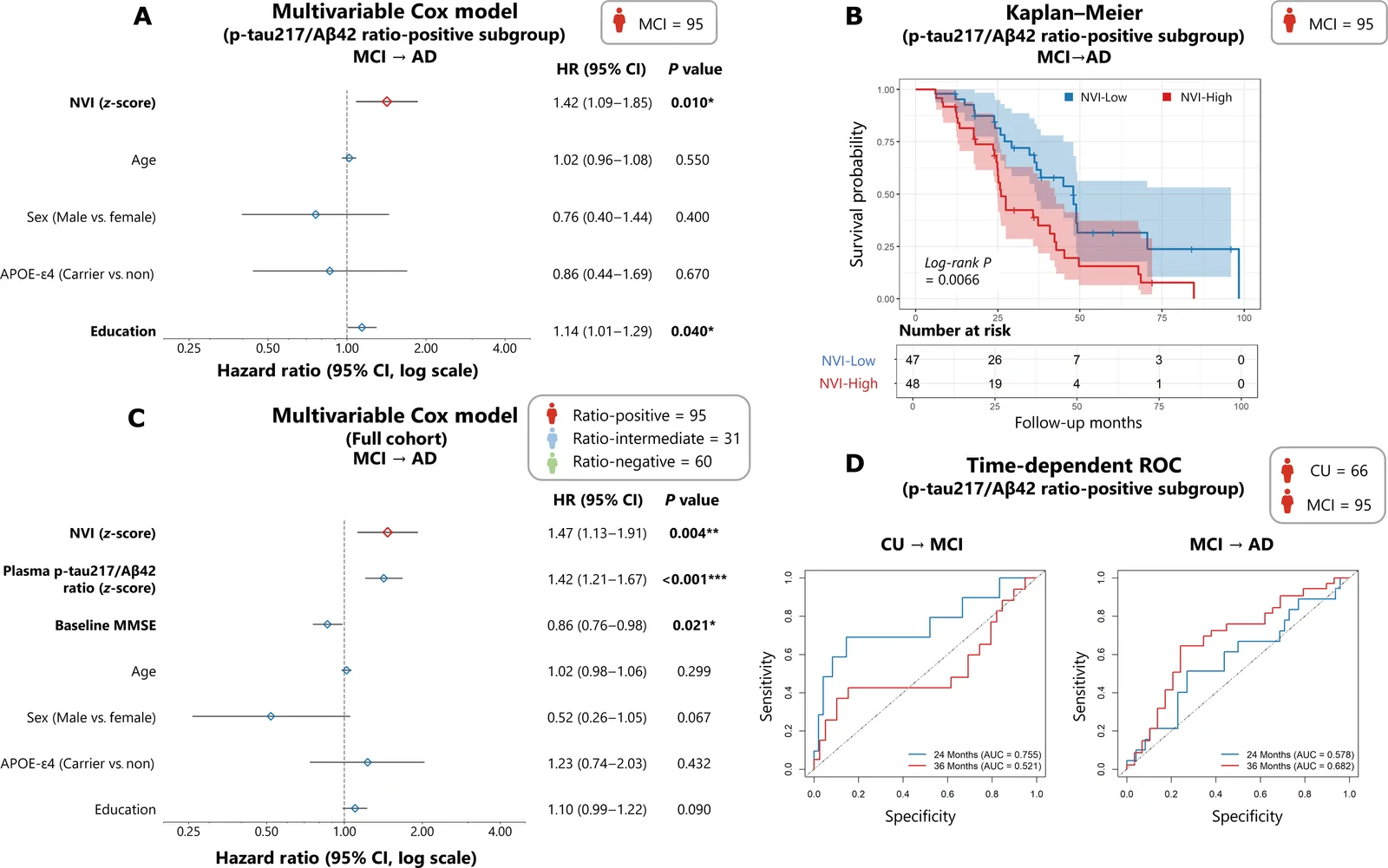
Longitudinal prognostic utility of the Network Vulnerability Index (NVI) for clinical progression. (A) Forest plot illustrating the hazard ratios derived from a Cox proportional hazards model for predicting the conversion from mild cognitive impairment (MCI) to Alzheimer’s disease (AD) dementia within the plasma-positive subcohort. The analysis is adjusted for age, sex, education, and *APOE* ε4 status. (B) Kaplan–Meier survival analysis estimating the progression-free probability from MCI to AD dementia. The plasma-positive subcohort was stratified into NVI-High (red line, high vulnerability) and NVI-Low (blue line, low vulnerability) groups based on the median NVI score. Shaded regions denote 95% confidence intervals, and the *P* value was calculated using the log-rank test. The accompanying risk table below the plot indicates the number of individuals remaining at risk at corresponding follow-up intervals. (C) Forest plot displaying the independent prognostic value of the NVI across the full cohort (encompassing the entire biomarker spectrum). This comprehensive Cox regression model evaluates the transition from MCI to AD dementia, strictly adjusting for continuous plasma phosphorylated tau (p-tau) 217/Aβ42 levels, age, sex, education, *APOE* ε4 status, and baseline Mini-Mental State Examination (MMSE) scores. (D) Time-dependent receiver operating characteristic (ROC) curves evaluating the temporal discriminative accuracy of the NVI for predicting longitudinal clinical conversion within the plasma-positive subcohort. The curves illustrate the predictive performance for the transition from cognitively unimpaired (CU) to MCI (left) and from MCI to AD dementia (right). The prognostic capacity is assessed at 24-month and 36-month follow-up intervals, with the corresponding area under the curve (AUC) values annotated to quantify the time-specific prediction accuracy.

The prognostic performance of the NVI exhibited a distinct, stage-dependent trajectory. During the clinical transition window from MCI to dementia, the NVI demonstrated robust and independent prognostic value. In the adjusted Cox model, each SD increase in the NVI was significantly associated with a 41.9% increased hazard of clinical progression (hazard ratio [HR] = 1.419, 95% confidence interval [CI]: 1.087 to 1.853, *P* = 0.010; Fig. 4A and Table S2). Consistent with this, Kaplan–Meier survival analysis revealed a significantly more rapid clinical decline in the NVI-High group compared to the NVI-Low group (log-rank test, *P* = 0.007; Fig. 4B). Time-dependent ROC analysis further confirmed its longitudinal discriminative capacity, yielding an AUC of 0.682 for predicting 36-month clinical conversion in this late preclinical stage (Fig. 4D). Conversely, for the earlier transition from CU to MCI, the overall Cox proportional hazard was not statistically significant (HR = 1.091, *P* = 0.602; Table S2). However, the NVI exhibited high discriminative power for predicting short-term precipitous decline, achieving a 24-month AUC of 0.755 (Fig. 4D).

### Incremental prognostic value of the NVI

Having established the stage-dependent utility of the NVI in extreme biomarker phenotypes (plasma p-tau217/Aβ42 ratio-positive), we next aimed to evaluate its generalized clinical applicability. To evaluate whether the neuroimaging-derived NVI provides additive prognostic utility beyond plasma biomarkers and clinical assessments across a continuous disease spectrum, we performed hierarchical Cox survival analyses across the extended longitudinal MCI cohort (*n* = 186, comprising 69 total conversion events over a maximum follow-up of 123 months). Crucially, this comprehensive analysis reincorporated participants across the entire biomarker continuum, including biomarker-negative (*n* = 60), intermediate (*n* = 31), and biomarker-positive (*n* = 95) individuals. Specifically, reintegrating the intermediate group (*n* = 31, accounting for 11 incident conversion events) not only maximized our statistical power but also accurately simulated a real-world clinical screening setting where ambiguous fluid biomarker profiles are frequently encountered.

During the clinical transition window from MCI to dementia, the base model, strictly adjusting for continuous plasma p-tau217/Aβ42 levels, age, sex, education, *APOE*-ε4, and baseline MMSE scores, yielded a baseline prognostic concordance index (C-index) of 0.724 (events per variable [EPV] = 9.86). Crucially, the incorporation of the continuous NVI into the full model significantly improved the overall predictive performance (likelihood ratio test, $\chi^{2}$ = 8.369, *P* = 0.003), increasing the C-index to 0.749. In this fully adjusted model, higher baseline NVI remained a strong independent predictor of clinical progression (HR = 1.473, 95% CI: 1.134 to 1.912, *P* = 0.004; Fig. 4C). These data collectively demonstrate that the spatial topography of DMN–limbic vulnerability offers essential, incremental prognostic information for AD progression.

## Discussion

In this study, we combined plasma biomarker-defined risk stratification with network-based epicenter mapping to identify spatially organized vulnerability patterns across the AD continuum. Individuals positive for the p-tau217/Aβ42 ratio exhibited highly reproducible neuroanatomical epicenters anchored along the DMN–limbic axis. Regionally, baseline epicenter centrality predicted future longitudinal atrophy rates. Individually, our validated NVI robustly tracked cross-sectional cognition and core AD pathology across cohorts, while independently forecasting multistage clinical conversion to provide incremental prognostic value beyond plasma assays alone. Coupled with multiscale alignments linking epicenter vulnerability to specific transcriptomic, neurotransmitter, and cognitive architectures, these findings support a framework in which pathological risk indexed by plasma p-tau217/Aβ42 can be spatially contextualized within connectome-based neurodegenerative epicenters, providing a scalable, clinically actionable neuroimaging refinement to blood-based prescreening strategies.

A key finding was the convergence of these p-tau217/Aβ42 ratio-defined epicenters onto the DMN–limbic axis. Specifically, our mapping identified a vulnerable network configuration spanning canonical cortical and deep gray matter structures, including the medial prefrontal cortex, posterior cingulate/precuneus, hippocampus, and amygdala. This topographic distribution strongly echoes recent cross-disorder connectome modeling [19], which implemented the epicenter framework to identify bilateral hippocampal regions as primary nodes of vulnerability across the symptomatic AD and progressive mild cognitive impairment spectrum. Crucially, this structural convergence provides robust mechanistic support for the view that highly integrated hubs, by virtue of their central connectome position, serve as plausible anchors for network-constrained degeneration, with tau accumulation and glial responses aligning to macroscale connectivity gradients that traverse these systems [22]. Indeed, both early amyloid deposition and tau burden have been shown to concentrate in DMN hubs [23,24], and whole-brain functional connectivity (FC) accurately predicts regional tau-PET distribution [25,26].

The biological relevance of the DMN–limbic epicenter is further supported by convergence across scales. Transcriptomically, its profile suggests an inverse coupling between synaptic signaling and energy metabolism, echoing recent quantitative functional magnetic resonance imaging (fMRI) evidence of discordant neurovascular coupling specifically within the DMN [27] and is in line with tau’s interactome links to synaptic and mitochondrial machinery [28–30]. Importantly, given the modest proportion of variance explained by the transcriptomic components, these molecular inferences should be treated strictly as hypothesis-generating frameworks. Their biological interpretation relies fundamentally on spatial alignments and existing literature rather than direct mechanistic evidence. Neurotransmitter maps indicate that epicenters tend to fall in regions with relatively lower dopaminergic, noradrenergic, and serotonergic receptor densities; coupled with brainstem-to-cortex gradients, this aligns with evidence implicating the locus coeruleus as an early hub of tau pathology [31,32]. Functionally, the map aligns positively with cognitive domains lost early in AD (for example, episodic and semantic memory) and negatively with frontoparietal control and sensorimotor functions (for example, executive control and planning) [33,34]. Together, these convergences provide a molecular and functional rationale for the selective vulnerability of the DMN–limbic axis, as captured by our plasma p-tau217/Aβ42 ratio-defined model.

The functional relevance of this baseline epicenter map is strongly supported by our longitudinal atrophy analysis. We found that regions identified as epicentral at baseline, particularly within DMN–limbic circuits, underwent the most rapid subsequent structural decline. This spatiotemporal link aligns with the network degeneration hypothesis [17,35,36], which posits that atrophy propagates along existing structural and functional connections, emerging first in regions most strongly linked to the epicenter [37]. This finding is also consistent with evidence that baseline tau-PET topography predicts the spatial pattern of future atrophy [21,38]. Furthermore, research indicates that tau propagation does not follow a single path, but rather multiple spatiotemporal trajectories [39]. Our individualized, connectome-constrained approach may therefore capture this progression heterogeneity in disease evolution beyond uniform staging models.

While foundational epicenter models have predominantly functioned as group-level explanatory tools, our study translates this spatial theory into a personalized prognostic utility via the derivation and external validation of the NVI. Although localized measures such as hippocampal volume serve as robust proxies for site-specific downstream injury and effectively capture the neurodegenerative “N” in the A/T/N framework [40], they inherently overlook the topological pathways through which pathology propagates. The NVI addresses this limitation by quantifying how an individual’s whole-brain gray matter variation aligns with the spatial constraints of a network diffusion process. Our cross-sectional data support this comprehensive capacity. Although the NVI was strongly correlated with traditional hippocampal volume loss (*r* = −0.455), it simultaneously captured a broader topographic profile tightly linked to entorhinal tau-PET accumulation (*r* = 0.405) and meta-temporal tau spread (*r* = 0.547). This specific link to tau pathology supports the model that tau spreading occurs in a stepwise fashion through functional networks [41,42]. Collectively, these multimodal correlations confirm that our network metric yields not merely a mathematical abstraction but a biologically grounded index of structural and molecular vulnerability.

Most importantly, the extended spatial context captured by the NVI translates into tangible, stage-dependent predictive power. Our survival analyses revealed that the prognostic efficacy of the NVI is highly sensitive to the transition from MCI to AD dementia. The rapid clinical conversion observed in NVI-high individuals likely reflects the exhaustion of cognitive reserve and the irreversible structural collapse of the DMN–limbic network. As prior works suggest [18,43], highly integrated, metabolically active networks may facilitate resilience in health but become a liability in disease. During the early preclinical stage, compensatory functional network reorganization may temporarily mask structural decline; however, once the disease progresses to the MCI stage, this compensation fails, and the macroscopic network vulnerability quantified by the NVI directly dictates the speed of cognitive deterioration.

Furthermore, the clinical utility of the NVI manifests dynamically depending on the target population. Within the strictly plasma-positive subcohort, the NVI serves as an effective downstream staging tool, differentiating individuals with stable molecular risk from those at imminent risk of clinical decline. Conversely, when evaluated across the full cohort encompassing the entire biomarker spectrum, the continuous NVI significantly improved prognostic accuracy beyond baseline clinical scores (MMSE) and absolute plasma p-tau217/Aβ42 levels. This distinction is critical: While fluid biomarkers reliably detect the presence and molecular severity of AD pathology, the NVI captures the individual’s topological proximity to the dementia threshold, proving that structural network integrity provides incremental prognostic value independent of molecular load.

While plasma p-tau217/Aβ42 captures systemic molecular risk, it lacks spatial resolution. Our structural NVI provides this crucial spatial anchor by mapping person-specific neurodegeneration onto a normative connectome. Existing literature strongly supports multimodal approaches, demonstrating that blood markers generalize better and predict clinical conversion more accurately when paired with spatial information from neuroimaging [44,45]. Head-to-head comparisons already suggest that the joint use of plasma p-tau217 and tau-PET outperforms either modality alone [46]. Together, our findings prospectively support a scalable “plasma pre-screen + structural imaging refinement” pathway. In this 2-step triage workflow, high-throughput plasma prescreening identifies at-risk individuals [47,48], followed by NVI calculation from routine T1-weighted magnetic resonance imaging (MRI) to refine individualized prognosis and trial enrichment without the logistical burden of widespread PET. Importantly, deriving the NVI incurs no additional cost over assessing established focal biomarkers like hippocampal volume. Rather than superseding these conventional metrics, the NVI leverages the exact same standard scan to offer a complementary, network-level perspective that topographically contextualizes localized limbic atrophy.

This study has several limitations. First, while validated across 2 distinct cohorts (primarily White/Caucasian in ADNI and Asian/Han Chinese in C-ADNI), the absence of other demographic groups (e.g., African American and Hispanic/Latino) warrants validation in more diverse populations. Second, the p-tau217/Aβ42 cutoffs utilized here were cohort- and assay-specific; rigorous cross-platform standardization is necessary before clinical translation. Third, our cross-modal transcriptomic mapping relies on the Allen Human Brain Atlas. This atlas features a highly restricted sample size and predominant left-hemisphere coverage, making the macro-to-micro spatial alignments susceptible to registration errors and highly dependent on upstream preprocessing choices. Fourth, the derivation of the NVI via LASSO regression assumes fundamentally linear relationships. Although this data-driven approach yields an interpretable sparse signature, it may overlook more complex, nonlinear spatiotemporal dynamics of network degeneration. Finally, our framework focused primarily on amyloid and tau pathways, omitting other key AD-related copathologies, such as neuroinflammation or cerebrovascular disease, which may modulate network vulnerability and contribute to individual differences in resilience and disease progression.

In conclusion, this study demonstrates that the systemic molecular risk defined by the plasma p-tau217/Aβ42 ratio can be robustly anchored to specific, connectome-constrained topographies of neurodegeneration. By identifying reproducible epicenters along the DMN–limbic axis, our findings provide empirical validation for the role of these highly integrated hubs as primary conduits for pathological propagation. Crucially, by translating this spatial blueprint into the individualized NVI, we provide a biologically grounded metric that captures the topological progression of the disease. Ultimately, this framework supports an actionable “plasma pre-screen + structural imaging refinement” pathway, offering a highly scalable strategy for early risk tiering and individualized prognostication. Future work should prioritize cross-assay standardization, validation in multiethnic cohorts, and the integration of patient-specific connectomics to further advance precision network medicine in AD.

## Materials and Methods

### Study population

Our analysis was conducted on a primary discovery cohort (ADNI) and subsequently replicated in an independent validation cohort (C-ADNI). Additionally, resting-state fMRI data from the Human Connectome Project (HCP) were utilized to construct a normative FC template.

### Discovery cohort (ADNI)

The ADNI was launched in 2003 as a public–private partnership, led by Principal Investigator Michael W. Weiner, MD. The primary goal of ADNI has been to test whether serial MRI, PET, other biological markers and clinical and neuropsychological assessment can be combined to measure the progression of MCI and early AD. Data were obtained from the ADNI (adni.loni.usc.edu). The ADNI study was approved by all local institutional review boards, and written informed consent was obtained from all participants or their authorized representatives. Participant selection was anchored to the availability of plasma biomarkers. We screened the ADNI UPENN_PLASMA_FUJIREBIO_QUANTERIX dataset (updated 2025 March 10) and identified 678 participants who had valid plasma p-tau217 and Aβ42 measurements at either the screening (“sc”) or baseline (“bl”) visits (defined by VISCODE2). This initial pool encompassed participants from ADNI Phases 1, GO, 2, 3, and 4. After excluding participants with intermediate plasma p-tau217/Aβ42 ratios or missing baseline T1-weighted MRI data, a final cohort of 573 participants was included in the main analysis (Table S3).

For these individuals, we retrieved and matched their corresponding baseline demographic information, *APOE* genotype, cognitive assessments (CDR-SB, MMSE, and MoCA) and all available baseline and longitudinal raw T1-weighted MRI scans. Additionally, we retrieved standardized, ADNI-provided, quality-controlled derivatives to ensure comparability across scanners and phases. Specifically, we assembled: (a) amyloid PET Centiloid values from the UC Berkeley 6-mm analysis tables, used as a tracer-harmonized global Aβ burden; (b) tau PET standardized uptake value ratios (SUVRs) from the UC Berkeley 6-mm partial-volume-corrected (PVC) release, using 2 regional summaries—the meta-temporal composite and entorhinal cortex; (c) regional GMVs from the UCSF Cross-Sectional FreeSurfer 7.x dataset, from which bilateral hippocampal volume was defined as ST29SV (left) + ST88SV (right); and (d) intracranial volume (ICV), primarily estimated intracranial volume from the University of California, San Diego volumetry (UCSDVOL) table with quality control-pass entries, supplemented by ICV from the Penn Image Computing and Science Laboratory / Automatic Segmentation of Hippocampal Subfields high-resolution T2 dataset when estimated intracranial volume was unavailable. For each participant, derivatives were time-aligned to the screening/baseline visit (VISCODE2 = “sc”/“bl”); when a baseline PET was not available, we selected the nearest exam within 24 months of baseline (favoring the closest visit) and recorded the PET–baseline month offset for use as a covariate. All merges used participant identification numbers, with VISCODE2 ensuring visit matching.

### Validation cohort (C-ADNI)

An independent validation sample (*n* = 340) was recruited from C-ADNI between 2016 and 2025, centrally coordinated at Nanjing Drum Tower Hospital. The study was approved by the Institutional Review Board of Nanjing Drum Tower Hospital, and all participants provided written informed consent prior to participation. Inclusion criteria for this analysis were as follows: age 55 to 80 years, right-handedness, ≥8 years of education, the availability of both plasma p-tau217/Aβ42 data and a high-resolution T1-weighted MRI scan. Exclusion criteria included cerebrovascular disease (e.g., stroke), major psychiatric disorders (e.g., severe depression or anxiety), other neurological diseases that could affect cognition (e.g., brain tumor, Parkinson’s disease, encephalitis, and epilepsy), or contraindications to MRI scanning. Demographic details and cognitive function test scores (MMSE and MoCA) were obtained for all participants. *APOE-ε4* carrier status was also assessed where available.

### Normative functional connectome (HCP)

To construct a high-quality normative FC template, we utilized resting-state fMRI data from the HCP S1200 release. From this release, we selected a random subset of 500 healthy young adults (age 22 to 35 years) to serve as a representative normative cohort. Data were acquired on a customized 3T Siemens Connectome Skyra scanner. We utilized data that had undergone the HCP minimal preprocessing pipelines and artifact removal using ICA-FIX. This normative connectome served as the reference for defining the healthy network architecture against which disease-related atrophy patterns were mapped.

### Plasma biomarker quantification and grouping

#### Discovery cohort (ADNI)

Plasma Aβ42 and p-tau217 levels were measured using the Lumipulse platform (for Aβ42) and the Simoa HD-X assay (for p-tau217), respectively. Detailed assay methodologies, platform specifics (Fujirebio and Quanterix) and quality control protocols are publicly documented by the ADNI Biomarker Core. These details can be found on the ADNI data archive (adni.loni.usc.edu) in the “Biospecimen” section, under the methods file titled “UPENN_PLASMA_FUJIREBIO_QUANTERIX”. Participants were stratified by their plasma p-tau217/Aβ42 ratio into negative (<0.0055) and positive (>0.0086) groups, applying the provisional “two-tier” cut points established by the ADNI Biomarker Core. These thresholds were derived from an ADNI-independent UPENN ADRC cohort and validated within the ADNI population, as detailed in the aforementioned methods document. To further substantiate the validity of this plasma biomarker stratification, we additionally performed a comparative ROC analysis evaluating the continuous plasma p-tau217/Aβ42 ratio versus p-tau217 alone against baseline Aβ-PET positivity. Individuals with intermediate values were excluded from the primary cross-sectional epicenter discovery to ensure optimal pathological contrast. This adheres to the Biomarker Core’s guidance, which defines this range as a zone of diagnostic uncertainty where amyloid status cannot be accurately determined. However, to maximize statistical power and simulate a continuous, real-world clinical setting, these intermediate participants were strategically reincluded in the subsequent full-cohort longitudinal survival models to evaluate the generalized prognostic utility of the derived imaging metrics.

#### Validation cohort (C-ADNI)

Plasma biomarkers were analyzed using blood samples from 242 participants in the validation cohort. All blood samples were collected between 9:00 AM and 11:00 AM. Within 1 h of collection, whole blood was centrifuged for 10 min; the resulting plasma was extracted and immediately stored at −80 °C in the Nanjing Drum Tower Hospital Biobank (Jiangsu Provincial Science and Technology Resources Coordination Service Platform). Prior to analysis, plasma samples were thawed at room temperature, fully mixed and then centrifuged at 1,000g and 4 °C for 5 min. Precipitates and the upper layer of floating debris were discarded, and the intermediate clear plasma layer was extracted for testing. Biomarker concentrations were quantified using the Simoa HD-X platform (Quanterix, Billerica, MA, USA). Specifically, plasma p-tau217 was measured using the Simoa p-tau217 V2 Assay Kit (Quanterix, LOT: 999024), while plasma Aβ42 was measured using the Simoa Neuro 4-Plex E Kit (Quanterix, LOT: 503940). Because the analytical platforms and calibrations differed between the discovery (ADNI: Lumipulse/Simoa) and validation (C-ADNI: Quanterix Simoa) cohorts, C-ADNI-specific cutoffs were derived using a 2-component Gaussian mixture model fitted to the p-tau217/Aβ42 ratio distribution. Furthermore, the C-ADNI subcohort was predominantly enriched for individuals in the earliest preclinical stages (e.g., subjective cognitive decline). In the absence of widespread baseline Aβ-PET imaging to serve as a diagnostic ground truth for this early-stage population, this unsupervised, data-driven approach is highly recommended to stratify pathological subgroups. As visually detailed in Fig. S7, the Gaussian mixture model effectively isolated the right-skewed pathological long tail from the dense normative peak. The intersection point of the 2 components, where the posterior probability of belonging to the high component was 0.5, was defined as the threshold separating the negative and positive subgroups (negative < 0.096; positive > 0.101). All plasma biomarker values were *z*-scored prior to analysis.

### Neuroimaging processing and epicenter mapping

#### Neuroimaging acquisition and preprocessing

High-resolution T1-weighted MRI data were acquired for all participants in both cohorts. For the ADNI cohort, structural brain images were acquired using 1.5 or 3.0T scanners with T1-weighted scans, typically using a sagittal volumetric magnetization-prepared rapid gradient echo sequence. Detailed imaging protocols are available at https://adni.loni.usc.edu/data-samples/adni-data/neuroimaging/mri/mri-scanner-protocols/. For the Validation cohort (C-ADNI), MRI scans were acquired on a Philips Ingenia CX 3.0T MR scanner with a 32-channel head coil using the following parameters: 196 sagittal slices; repetition time = 8.10 ms; echo time = 3.70 ms; field of view = 256 × 256 mm^2^; voxel size = 1 × 1 × 1 mm^3^; and slice thickness = 1 mm.

All acquired T1-weighted images were processed using an identical pipeline via the Computational Anatomy Toolbox (CAT12; www.neuro.uni-jena.de/cat/) within SPM12. Preprocessing steps included spatial normalization to Montreal Neurological Institute space, tissue segmentation (gray matter, white matter, and CSF), bias-field correction and modulation to preserve volumetric information. The resulting GMV images were smoothed with an 8-mm full-width at half-maximum Gaussian kernel.

#### Determination of individual gray matter reduction *z*-score map

To generate individualized maps of gray matter reduction, we followed the methodology established in prior epicenter mapping studies [19]. First, voxel-wise GMV maps for all participants were adjusted by regressing out the effects of age, the square of age, sex, total intracranial volume, and site using a general linear model within the gray matter mask. For each participant, their adjusted GMV map was normalized relative to the plasma p-tau217/Aβ42 ratio-negative group using a *z*-score procedure. This created a voxel-wise, individualized *z*-score map for each participant, where a more positive *z*-score represented a larger deviation from the control mean. To enhance sensitivity to meaningful atrophy, a spatial mask was applied to each participant’s *z*-score map, retaining only voxels with a *z*-score magnitude *z* > 0.5 for the subsequent epicenter analysis. To verify that the identified epicenter topography was not biased by this predefined cutoff, a comprehensive sensitivity analysis was additionally conducted by reevaluating the spatial consistency of the epicenter metrics across a continuous spectrum of alternative *z*-score thresholds (*z* > 0.3, 0.4, 0.6, and 0.7).

#### Characterization of the plasma p-tau217/Aβ42 ratio-positive epicenter map

To identify the plasma p-tau217/Aβ42 ratio-positive epicenter, we used patient-tailored, connectivity-based epicenter mapping. This method hypothesizes that an epicenter is a region whose intrinsic FC pattern best resembles the patient’s individual atrophy (*z*-score) pattern. First, a normative set of functional connectomes was derived from a HCP (*n* = 500). A whole-brain parcellation of 254 regions (200 cortical regions [49], 54 subcortical regions [50]) was used to define seed regions. For each of the 254 seeds, a group-level normative FC map was generated.

Next, for each participant in the plasma p-tau217/Aβ42 ratio-positive group, we computed a spatial Spearman correlation between their individual *z*-score map (masked at *z* > 0.5) and each of the 254 normative FC maps. This procedure yielded 254 GOF scores for each participant, corresponding to the 254 candidate epicenter regions. A higher GOF score indicated a stronger spatial similarity between the individual’s atrophy pattern and the region’s FC profile, suggesting that the region is a likely epicenter for that individual.

Finally, to determine the epicenters at the group level, a 1-sample *t* test was performed on the GOF scores for each of the 254 regions across all plasma p-tau217/Aβ42 ratio-positive participants. The resulting statistical *t*-map represented the inferred epicenter degree for all brain regions. To visualize these findings, we generated a Manhattan-like plot of epicenter *t* values across the 254 brain regions, organized according to their assigned network and subcortical structure partitions within the atlas. A comprehensive schematic diagram detailing the workflow of this section is provided in Fig. S8.

### Multiscale associations

#### Correlation analysis between epicenter and gene expression

We integrated the epicenter *t*-map with transcriptomic data from the Allen Human Brain Atlas. Regional gene expression was parcellated into the same 254 regions used in the imaging analysis. PLS regression was applied to assess spatial associations between epicenter *t* values and regional gene expression. Statistical significance of spatial associations was tested with spin permutation tests restricted to cortical regions (*n* = 5,000), and the stability of gene weights was assessed using bootstrap resampling (*n* = 5,000). A 2-sided *P*_spin_ < 0.05 was considered statistically significant. Genes were subsequently ranked according to their PLS loadings, and gene set enrichment analysis was conducted in a preranked framework across GO and Kyoto Encyclopedia of Genes and Genomes gene sets.

#### Correlation analysis between epicenter and neurotransmitter distribution

We assessed the spatial correspondence between the epicenter *t*-map and neurotransmitter receptor/transporter distribution. Nineteen receptor density maps, spanning 9 neurotransmitter systems (dopamine, norepinephrine, serotonin, acetylcholine, glutamate, GABA, histamine, cannabinoid, and opioid), were obtained from a large-scale PET-based atlas (Hansen et al., 2022; https://github.com/netneurolab/hansen_receptors). Each map was parcellated into 200 cortical regions using the same atlas as for the epicenter map. Spatial associations were quantified using Spearman correlations, with spin permutation tests applied to control for spatial autocorrelation (*n* = 5,000; *P*_spin_ < 0.05) and FDR correction applied for multiple comparisons (*q* < 0.05).

#### Correlation analysis between epicenter and cognitive functions

Meta-analytic task association maps for 115 cognitive terms were obtained from Neurosynth. All maps were parcellated to the same 254-region atlas used for the epicenter analysis, yielding a region-by-term matrix. We applied PLS regression to assess spatial associations between the epicenter *t*-map and the cognitive association matrix. Spatial autocorrelation was controlled using cortex-restricted spin permutation tests (*n* = 5,000; *P*_spin_ < 0.05). To evaluate the stability and contribution of cognitive terms to the significant PLS component, we computed bootstrap-based *z*-scores of term weights and applied FDR correction (*q* < 0.05).

### Statistical analysis

#### Epicenter map validation

To validate the spatial robustness of the predictive epicenter network, multiple sensitivity analyses were conducted. First, to assess cohort generalizability, the identical analytic framework was applied to the independent C-ADNI cohort to generate a validation epicenter *t*-map. Epicenter *t* values derived from ADNI and C-ADNI were compared using spatial Spearman correlations, with cortical-level significance tested using spin permutations (*n* = 5,000). The overlap between the top-ranked (top 5, 10, and 30) cortical and subcortical epicenters across cohorts was also quantified.

Second, to ensure that the findings were independent of the specific spatial parcellation scheme, the epicenter identification process was replicated using the Brainnetome Atlas.

Finally, to account for potential physiological network dedifferentiation during normal aging and to confirm robustness to the reference connectome, we conducted a template sensitivity analysis. An alternative, age-matched elderly normative connectome was constructed using data from CU, p-tau217/Aβ42 ratio-negative individuals within the C-ADNI cohort (*N* = 73). The regional GOF scores and corresponding epicenter t-maps were recomputed using this elderly reference network. The consistency of the resulting spatial topography was then cross-validated against the primary HCP-derived map via whole-brain spatial correlation analysis.

#### Longitudinal atrophy dynamics

Longitudinal structural analyses were restricted to the ADNI discovery cohort, as the C-ADNI validation cohort is currently cross-sectional by design. In the ADNI cohort, a total of 444 participants with at least 1 follow-up T1-weighted MRI scan were included. All baseline and follow-up T1-weighted MRI scans (mean 5.04 ± 2.60 scans per participant; mean follow-up 51.17 ± 36.51 months) were processed through the same VBM and general linear model pipeline described in the “Multiscale associations of neuroimaging epicenters” section. For each participant, adjusted GMV maps were derived after regressing out age, sex, the square of age, total intracranial volume, and site effects. Mean adjusted GMV values were then extracted for each of the 254 regions of interest (ROIs) at every available time point.

ROI-wise longitudinal change in GMV was estimated for each participant using linear regression of GMV residuals against time (using all available time points). The resulting regression coefficient (slope) was scaled to annualized units (ΔGMV/year). To visualize atrophy at fixed intervals, group comparison maps (p-tau217/Aβ42 ratio-positive vs. p-tau217/Aβ42 ratio-negative) were generated using data from participants with scans available at the 12-month (*n* = 441), 24-month (*n* = 358), and 36-month (*n* = 314) time points, respectively. Finally, to test whether baseline epicenter vulnerability predicts subsequent structural decline, we computed Pearson correlations between the group-level epicenter t-map (derived from the p-tau217/Aβ42 ratio-positive group at baseline) and the mean annualized atrophy slope map across ROIs, separately for cortical and subcortical regions. *P* < 0.005 was considered statistically significant.

#### Data-driven derivation and cross-cohort validation of NVI

To overcome the potential selection bias of arbitrary topological thresholding (e.g., selecting the top *N* regions) and to formulate a personalized vulnerability metric, we implemented a data-driven machine learning framework. Within the p-tau217/Aβ42 ratio-positive ADNI discovery cohort, a LASSO regression model was employed. Through 10-fold cross-validation, the model penalized collinearity and objectively selected regional GOF features to predict baseline cognitive impairment (defined as MMSE residuals, adjusted for age, sex, education and *APOE*-ε4 carrier status).

Based on the nonzero *β* coefficients derived from the optimized LASSO model, we formulated the NVI. For each individual, the NVI was calculated as the linear combination of their whole-brain regional GOF scores weighted by the LASSO-derived coefficients. The final sum was multiplied by −1 to ensure clinical interpretability (i.e., a higher NVI value indicates greater spatial vulnerability).

To test external generalizability, a strict out-of-sample validation was performed. The exact computational pipeline and model parameters derived from ADNI (including standard scalers, confounder regression weights, and the spatial LASSO coefficients) were frozen and applied directly to the independent C-ADNI cohort to predict clinical cognitive residuals. Generalization to other cognitive domains was further evaluated by assessing partial Pearson correlations between the NVI and CDR-SB, MoCA, and MMSE scores in both cohorts, adjusting for age, sex, education, and *APOE*-ε4 carrier status. *P* values were adjusted for multiple comparisons using the FDR procedure. Significance was defined as an FDR-adjusted *P* < 0.05 (*P*_FDR_ < 0.05).

#### Cross-sectional biological validation of NVI

To elucidate the biological underpinnings of the NVI, partial Pearson correlations were conducted between the individual NVI and established structural and molecular biomarkers at baseline. Importantly, these cross-sectional analyses were strictly restricted to the plasma p-tau217/Aβ42 ratio-positive subcohort. To maximize statistical power, samples were adaptively filtered based on the baseline availability of each specific neuroimaging modality. For analyses involving multiple neuroimaging endpoints, *P* values were adjusted for multiple comparisons using the FDR procedure. Significance was defined as an FDR-adjusted *P* < 0.05 (*P*_FDR_ < 0.05).

Hippocampal volume: Associations between NVI and bilateral hippocampal volume (*n* = 216) were assessed, controlling for age, sex, years of education, *APOE*-ε4 carrier status, MRI visit month, and ICV.

Tau-PET: Associations between NVI and tau-PET SUVRs (*n* = 151) were evaluated across specific topographical regions (entorhinal, inferior temporal, and a meta-temporal composite). We used partial-volume-corrected SUVRs to reduce spillover from atrophy and CSF. The hippocampus was not analyzed because off-target signal from the choroid plexus can bias hippocampal SUVRs. All tau-PET analyses controlled for age, sex, years of education, *APOE*-ε4 carrier status, and tau-PET visit month.

Aβ-PET: Associations between NVI and global Aβ Centiloid values (*n* = 192) were tested, controlling for age, sex, education, *APOE*-ε4 carrier status, and tracer indicator.

#### Survival analysis and temporal predictive accuracy for clinical conversion

All longitudinal analyses were restricted to participants with at least 1 follow-up assessment. To evaluate the prognostic utility of the neuroimaging-derived NVI within high-risk individuals, longitudinal time-to-conversion analyses were conducted within the plasma p-tau217/Aβ42 ratio-positive subcohort. Two clinical transitions were examined separately: CU →MCI and MCI → AD dementia. Time to event was defined as days from baseline diagnosis to first conversion or last available follow-up.

Multivariate Cox proportional hazards models were constructed for each conversion type. To prevent overfitting and adhere to the EPV statistical guidelines, the models incorporated only 5 predefined parameters: the standardized NVI (*z*-scored) and 4 essential covariates (age, sex, education, and *APOE*-ε4 status). The proportional hazards assumptions were verified using Schoenfeld residuals. HRs with 95% CIs were reported to reflect the increased risk of clinical progression per 1 SD increase in the NVI.

To further assess the temporal predictive accuracy of the model, time-dependent ROC curve analyses were performed using the linear predictors from the Cox models, calculating the AUC for 24-month and 36-month conversion horizons. Finally, Kaplan–Meier survival curves were plotted to visualize progression-free probabilities, with participants stratified into NVI-High and NVI-Low risk groups based on the median NVI score, and statistical differences were assessed via log-rank tests.

#### Incremental prognostic utility of NVI

To evaluate the additive clinical value of the neuroimaging-derived NVI beyond plasma biomarkers and standard cognitive assessments, we conducted an incremental prognostic analysis across the full cohort. Specifically, the LASSO weights derived from the high-risk (p-tau217/Aβ42 ratio-positive) discovery group were projected onto the entire spectrum of participants to calculate their individual NVI scores. Applying these predefined weights to the broader cohort effectively prevented statistical circularity while expanding the number of available clinical conversion events, thereby allowing for comprehensive covariate adjustment without violating the EPV guideline.

Hierarchical Cox proportional hazards models were subsequently constructed to predict longitudinal clinical progression. The base model incorporated the continuous plasma p-tau217/Aβ42 ratio, age, sex, education, *APOE*-ε4 carrier status, and baseline MMSE scores. The full model included all base covariates plus the individual NVI. The incremental predictive performance was quantified by the absolute change in Harrell’s C-index, and the significance of the overall improvement in model fit was statistically determined using the likelihood ratio test.

#### Statistical software and packages

All statistical analyses were performed in Python (version 3.11.5). Survival analyses, including multivariate Cox proportional hazards modeling, Kaplan–Meier estimations, log-rank tests, and C-index calculations, were implemented using the lifelines package (version 0.30.3). Feature selection via LASSO regression and time-dependent ROC evaluations were conducted using scikit-learn (version 1.8.0). Additional statistical tests, including likelihood ratio tests, utilized statsmodels (version 0.14.5) and SciPy (version 1.17.1).

## Data Availability

ADNI data are available from the ADNI data portal (https://adni.loni.usc.edu/). HCP Young Adult data are available from ConnectomeDB (https://www.humanconnectome.org/) and the NIMH Data Archive (https://nda.nih.gov/ccf/hcp). C-ADNI data are available from the study investigators upon reasonable request and with institutional approvals. The analysis code is publicly available at https://github.com/Jiamingglyy/neuroepicenter-gof.

## Supplementary Materials

Supplementary 1 Figs. S1 to S8 Tables S1 to S3 Supplementary_File_ADNI_Acknowledgement_List
