# Supplementary material for: Neuroimaging Epicenters as Vulnerable Nodes in Plasma p-tau217/Aβ42-Positive Alzheimer’s Disease
Source: Research (Wash D C). 2026 Aug 24;9:1392. doi: 10.34133/research.1392 (PMC13500913; doi:10.34133/research.1392)
Supplement: Supplementary file 1 [file research.1392.f1.zip › Supplementary files.docx]

Figures S1 to S8

**Supplementary Figure S1.** Diagnostic performance of the plasma p-tau217/Aβ42 ratio for detecting cerebral amyloidosis. Receiver operating characteristic (ROC) curve validating the continuous plasma p-tau217/Aβ42 ratio against baseline Aβ-PET positivity (*n* = 582; defined by the AMYLOID_STATUS column from the ADNI UCBERKELEY_AMY_6MM_03Dec2025.csv dataset). The assay demonstrated excellent diagnostic accuracy (AUC = 0.910), achieving 80.9% sensitivity and 89.9% specificity at the optimal cutoff. These results robustly confirm the pathological validity of the applied ratio-based clinical stratification.

**Supplementary Figure S2.** Sensitivity analysis of epicenter mapping across alternative voxel-selection thresholds. Scatter plots illustrate the spatial consistency of the regional epicenter degree derived from the primary threshold (z > 0.5) versus alternative thresholds (z > 0.3, 0.4, 0.6, and 0.7). Spearman’s rank correlation coefficients (*ρ*) and corresponding *P* values demonstrate exceptionally high spatial reproducibility (all *ρ* > 0.994, *P* < 0.001). These results indicate that the topographical distribution of the identified epicenters is robust and essentially independent of the specific mathematical threshold employed for voxel inclusion.

**Supplementary Figure S3.** Spatial robustness of the predictive epicenter network. To ensure that the identified epicenter patterns were not driven by the specific anatomical boundaries of the primary parcellation (Schaefer 200 and Tian 54), the epicenter mapping framework was strictly replicated using the Brainnetome Atlas (BNA). This figure displays the comparison of the group-level baseline epicenter t-maps derived from both the discovery cohort (ADNI) and the independent validation cohort (C-ADNI). Left panels exhibit the results from the primary parcellation, while right panels show the BNA replication. The topographical distribution of the t-values remains highly consistent across both atlases and cohorts, reaffirming that the focal vulnerability within the neocortical and deep grey matter structures (specifically characterizing the DMN-limbic network axis) is biologically robust and entirely independent of the chosen spatial resolution or parcellation scheme.

**Supplementary Figure S4.** Robustness of epicenter spatial topography to alternative age-matched reference connectomes. (A) Spatial distribution of the predictive epicenter t-map derived using the primary young adult reference connectome (HCP; ages 22–35). (B) Spatial distribution of the epicenter t-map derived using a cohort-specific normative connectome (C-ADNI; cognitively unimpaired, plasma p-tau217/Aβ42 ratio-negative older adults, *n* = 73). While physiological aging and network dedifferentiation naturally induce a shift in absolute alignment scores, the relative vulnerability pattern remains highly consistent. (C) Scatter plot demonstrating the spatial correlation across the whole brain between the epicenter t-maps derived from the two distinct reference templates. The highly significant positive association (Spearman *ρ* = 0.410, *P* < 0.001) confirms that the fundamental macroscale topography of the predictive epicenters is highly conserved, independent of the reference template’s age.

**Supplementary Figure S5**. Multiscale biological convergence of the epicenter map. **(A)** Gene set enrichment analysis (GSEA) of Gene Ontology (GO) biological processes (top) and KEGG pathways (bottom). Bar lengths represent the normalized enrichment scores (NES), and the color gradient indicates statistical significance (-log_10_*P_FDR_*). The first partial least squares component (PLS1) displays a bipolar transcriptomic signature: the negative-loading pole is enriched for excitatory postsynaptic density and glutamatergic receptor–related processes, whereas the positive-loading pole is enriched for mitochondrial respiratory chain, oxidative phosphorylation complexes and fatty-acid β-oxidation. **(B)** Correlations between the epicenter map and 19 in-vivo neurotransmitter PET maps. *: *P*_spin_ < 0.05. D_2_: dopamine D_2_, NAT: noradrenaline/norepinephrine transporter, 5-HT_1b_: serotonin 5-HT_1b_. **(C)** Correlations with 115 cognitive function maps. Bars show the top 10 positive and top 10 negative loadings. Positive loadings (higher epicenter values) cluster in memory and socio-emotional processes; negative loadings cluster in executive and motor control domains.

**Supplementary Figure S6.** Longitudinal atrophy maps of deep grey matter structures. To prevent surface-projection artifacts and accurately represent deep grey matter structures, subcortical statistics are displayed using axial mosaic slices.

**Supplementary Figure S7.** **Data-driven threshold derivation for the plasma p-tau217/Aβ42 ratio in the C-ADNI cohort using a Gaussian Mixture Model.** Characteristic of early-stage preclinical populations, the empirical plasma ratio exhibits a heavily right-skewed distribution. A two-component Gaussian mixture model distinguishes the dense normal peak (blue line) from the pathological long tail (red line). The derived positivity threshold (> 0.101, dotted line) corresponds to the intersection where the posterior probability reaches 0.5, objectively isolating the high-risk subgroup.

**Supplementary Figure S8.** Methodology schematic for patient-tailored, connectivity-based epicenter mapping of p-tau217/Aβ42 ratio-positive AD. This figure illustrates the pipeline used to identify the probable neuroanatomical origins (epicenters) of brain atrophy associated with p-tau217/Aβ42 ratio-positive AD pathology. (A) Study population and neuroimaging data. T1-weighted structural MRI scans were obtained from a total of N=528 individuals, categorized based on their plasma p-tau217/Aβ42 ratio into Ratio-Positive patients (N=216, indicated in orange tones) and Ratio-Negative cognitively unimpaired controls (N=312, indicated in blue tones). (B) Standardized image processing. Structural MRI data underwent standard Voxel-Based Morphometry (VBM) preprocessing pipelines. Voxel-wise Grey Matter Volume (GMV) maps were generated. Confounding effects of age, sex, total intracranial volume (TIV), and scanner site were regressed out using a General Linear Model (GLM), followed by applying a grey matter mask. (C) Individual atrophy map generation. A normative model of grey matter distribution was constructed using the Ratio-Negative group as the healthy reference. For each Ratio-Positive individual, a voxel-wise z-score map was calculated to quantify the deviation of their GMV from the normative mean. A higher positive z-score indicates greater atrophy severity relative to the Ratio-Negative controls. (D) Determining individual epicenter Goodness-of-Fit (GOF) scores. This step tests the hypothesis that atrophy patterns reflect disease spread along healthy functional network architecture. Functional Connectivity (FC) maps for 254 predefined seed regions (atlas ROIs) were derived from an independent healthy cohort (Human Connectome Project). For a given patient, spatial correlation was computed between their individual atrophy z-score map and the healthy FC map of each seed region. This correlation coefficient serves as the "Goodness-of-Fit" (GOF) score. A high GOF score for a specific seed region indicates that atrophy in the patient is preferentially concentrated in areas functionally coupled to that seed, identifying the seed as a likely pathological epicenter. (E) Group-level epicenter mapping. The resulting 254 GOF scores for each individual were collated. One-sample t-tests was performed across the Ratio-Positive group to identify regions with significantly consistent high GOF scores, resulting in a group-level t-map highlighting the most probable structural epicenters of the disease pathology.

Tables S1-3

**Supplementary Table S1.**

**Regional feature weights of the Network Vulnerability Index derived from LASSO regression based on the combined Schaefer and Tian parcellations.**

| ROI | Weight | Label | Network |
| --- | --- | --- | --- |
| 199 | 2.4155 | 7Networks_RH_Default_pCunPCC_2 | Default |
| 157 | 1.7274 | 7Networks_RH_SalVentAttn_Med_2 | SalVentAttn |
| 152 | 1.6285 | 7Networks_RH_SalVentAttn_FrOperIns_1 | SalVentAttn |
| 69 | 1.4496 | 7Networks_LH_Cont_PFCl_4 | Cont |
| 60 | 1.4008 | 7Networks_LH_Limbic_TempPole_4 | Limbic |
| 105 | 1.2949 | 7Networks_RH_Vis_5 | Vis |
| 211 | 1.201 | THA-DAm-rh | Basal_Ganglia |
| 162 | 1.1552 | 7Networks_RH_Limbic_TempPole_1 | Limbic |
| 247 | 0.9827 | CAU-tail-lh | Basal_Ganglia |
| 94 | 0.8297 | 7Networks_LH_Default_PFC_12 | Default |
| 57 | 0.7614 | 7Networks_LH_Limbic_TempPole_1 | Limbic |
| 131 | 0.6355 | 7Networks_RH_SomMot_16 | SomMot |
| 210 | 0.5407 | THA-VAs-rh | Basal_Ganglia |
| 68 | 0.4273 | 7Networks_LH_Cont_PFCl_3 | Cont |
| 226 | 0.2255 | pGP-rh | Basal_Ganglia |
| 227 | 0.2041 | aGP-rh | Basal_Ganglia |
| 93 | 0.033 | 7Networks_LH_Default_PFC_11 | Default |
| 171 | -0.0197 | 7Networks_RH_Cont_PFCl_2 | Cont |
| 160 | -0.0978 | 7Networks_RH_Limbic_OFC_2 | Limbic |
| 100 | -0.1094 | 7Networks_LH_Default_PHC_1 | Default |
| 79 | -0.1148 | 7Networks_LH_Default_Par_1 | Default |
| 189 | -0.4057 | 7Networks_RH_Default_Temp_5 | Default |
| 3 | -0.4416 | 7Networks_LH_Vis_3 | Vis |
| 154 | -0.4735 | 7Networks_RH_SalVentAttn_FrOperIns_3 | SalVentAttn |
| 182 | -0.5338 | 7Networks_RH_Default_Par_1 | Default |
| 242 | -0.5971 | PUT-VP-lh | Basal_Ganglia |
| 58 | -0.6667 | 7Networks_LH_Limbic_TempPole_2 | Limbic |
| 86 | -0.8112 | 7Networks_LH_Default_PFC_4 | Default |
| 188 | -0.8444 | 7Networks_RH_Default_Temp_4 | Default |
| 80 | -0.8537 | 7Networks_LH_Default_Par_2 | Default |
| 240 | -0.9798 | PUT-VA-lh | Basal_Ganglia |
| 250 | -1.1382 | THA-DP-lh | Basal_Ganglia |
| 186 | -1.1409 | 7Networks_RH_Default_Temp_2 | Default |
| 225 | -1.4046 | NAc-core-rh | Basal_Ganglia |
| 234 | -1.708 | HIP-tail-lh | Limbic |
| 5 | -1.9148 | 7Networks_LH_Vis_5 | Vis |
| 248 | -2.1005 | lAMY-lh | Limbic |

**Supplementary Table S2.**

**Cox models of progression in plasma p-tau217/Aβ42 ratio-positive individuals (ADNI).**

| **Conversion Type** | **Predictors** | **Hazard Ratio (HR)^a^** | **HR CI Lower** | **HR CI Upper** | ***P*** |
| --- | --- | --- | --- | --- | --- |
| **CU to MCI** |  |  |  |  |  |
| (N=66, Events=42) | NVI (per 1 SD) | 1.092 | 0.785 | 1.520 | 0.602 |
|  | **Age** | **1.091** | **1.023** | **1.164** | **0.008^**^** |
|  | Sex Female (vs. Male) | 1.054 | 0.515 | 2.158 | 0.886 |
|  | *APOE-ε4* Carrier (Yes vs. No) | 1.167 | 0.596 | 2.287 | 0.653 |
|  | Education | 0.998 | 0.863 | 1.154 | 0.977 |
| **MCI to AD** |  |  |  |  |  |
| (N=95, Events=53) | **NVI (per 1 SD)** | **1.419** | **1.087** | **1.853** | **0.010^**^** |
|  | Age | 1.017 | 0.962 | 1.076 | 0.550 |
|  | Sex Female (vs. Male) | 0.761 | 0.404 | 1.436 | 0.400 |
|  | *APOE-ε4* Carrier (Yes vs. No) | 0.864 | 0.442 | 1.692 | 0.670 |
|  | **Education** | **1.139** | **1.006** | **1.289** | **0.040^*^** |

*Note:* Analyses were restricted to the plasma p-tau217/Aβ42 ratio-positive sub-cohort. The Network Vulnerability Index (NVI) was Z-scored; thus, its hazard ratio (HR) reflects the risk associated with a 1-SD increase. Age and education are modeled per 1-year increment. ᵃ*HR* > 1 indicates increased conversion risk. * *P* ≤ 0.05, *P* ≤ 0.01. Abbreviations: CU, cognitively unimpaired; MCI, mild cognitive impairment; AD, Alzheimer’s disease; CI, confidence interval; SD, standard deviation.

**Supplementary Table S3.**

Participant Selection and Exclusion Criteria for the ADNI Discovery Cohort

| **Step** | **Description** | ***N* (Participants)** |
| --- | --- | --- |
| **1. Initial Pool QC** | Total participants with available plasma biomarker data  (p-tau217 & Aβ42) in the *UPENN_PLASMA_FUJIREBIO_QUANTERIX* file. | 678 |
| **2. Modality Check** | **Exclusion:**  Missing corresponding baseline T1-weighted MRI scan. | 669 |
| **3. Ratio Filtering** | **Exclusion:**  Plasma p-tau217/Aβ42 ratio in the "Intermediate" range (0.0055 ≤ Ratio ≤ 0.0086). | 573 |
| **4.Final Cohort** | - Ratio Negative (< 0.0055) | 312 |
|  | - Ratio Positive (> 0.0086) | 261 |

**Note:** Data were obtained from the Alzheimer’s Disease Neuroimaging Initiative (ADNI) database. QC = Quality Control.
